# Improving the punching capacity of footings using geocell, geogrid and granular soil replacement

**DOI:** 10.1038/s41598-024-81251-y

**Published:** 2025-04-01

**Authors:** Ahmed M. Ebid, Nada M. Abdelhamid, Amr H. Zaher, Dina M. Ors

**Affiliations:** 1 Structural Engineering and Construction Management Department, Future University in Egypt (FUE), New Cairo, Egypt; 2 Civil Engineering Department, Faculty of Engineering, Ain Shams University, Cairo, Egypt

**Keywords:** Punching capacity, Isolated footing, Contact pressure, Geogrid, Geocell, Civil engineering, Mechanical engineering

## Abstract

Punching failure in footings occurs in thin pads that is subjected to heavy concentrated loads, such as concrete pads below racks’ supports in heavy storage facilities. Since the punching capacity depends on the stress distribution beneath the footing, it could be enhanced by improving the soil stiffness beneath the footing. This research examines the effects of geocell layers, geogrid layers, and base soil stiffness on the punching capacity of isolated footings. Seven identical footings were subjected to experimental testing until failure, with continuous monitoring of load, settlement, strain in rebars, and contact pressure. The control footing was rested on pure sand; two footings were rested on one and two geogrid layers, two footings were rested on one and two geocell layers and the last two footings were rested on pure crouched stone and mixture of sand and crushed stone. The results indicated that all footings exhibited pure punching failure. In addition, enhancing the base granular soil stiffness through geocell confinement, geogrid reinforcement, or replacement with stiffer soil concentrated contact stress under the load, thereby increasing punching capacity, reducing settlement and increasing the subgrade reaction at the center while decreasing it at the mid-edge and corners. The efficiency of geocell and geogrid layers depends on their proximity to the footing; the closest geocell layer was most efficient, while the closest geogrid layer was least efficient. Implementing geocell layers, geogrid layers, and soil replacement improved punching capacity by 20%, 11%, and 17%; subgrade reaction values at the center by 90%, 65%, and 200%; and reduced settlement at the center by 70%, 75%, and 45%, respectively.

## Introduction

The punching shear capacity of reinforced concrete footings is a critical factor in structural engineering, yet there is significant variation in the capacity predictions provided by different design codes. These discrepancies largely stem from differences in how the soil reaction is accounted for in the punching load calculations across various codes^1^. Studies have indicated that the angle of the shear failure plane in footings is steeper compared to flat slabs, and that shear slenderness significantly affects punching shear capacity. Moreover, finite element analysis (FEA) has become an invaluable tool in understanding the behavior of reinforced concrete footings. Studies using FEA, calibrated with field test data, have shown good agreement with experimental results, though some challenges remain, particularly regarding the modeling of concrete crushing^2^. Zhang et al.^3^ conducted eccentric compression tests on three RC column footings, examining the complex soil-structure interaction. This study assessed the load-carrying capacity, displacement distribution, reaction distribution, failure modes, crack development, and strain distribution, with the primary test parameter being the flexural reinforcement ratio in the foundation slab. The results indicated that footings with lower reinforcement ratios were more prone to brittle punching shear failure, with the reinforcement ratio also affecting crack distribution and concrete cover spalling. The findings, however, should be interpreted with caution due to the limited number of specimens tested. A new method was proposed to predict the punching shear strength under eccentric compression, based on the test results and existing studies.

Previous research has often relied on experimental data derived from tests on slabs rather than actual footings. These experiments typically simulate soil conditions using springs, hydraulic jacks, or line supports, which do not fully replicate the behavior of soil in situ^4^. Recent research has aimed to address these issues by conducting both analytical and experimental investigations on footings supported on actual soil. This approach provides insights into how individual characteristics of footings and soil affect the punching shear capacity and how current design codes align with these observations^5^. The variation in design codes, such as ACI 318-19, Eurocode 2, and fib Model Code 2010, further complicates the prediction of punching shear capacity. These codes differ in their definitions of critical sections, handling of parameters such as shear slenderness and reinforcement ratios, and modeling of subsoil conditions^6^. For instance, the BS 8110-1:1997 is noted for its conservatism, while the second generation of the Eurocode (PrEC2) is considered more reliable and closer to experimental values. The undesired punching shear failure around columns is a critical issue for reinforced concrete flat plates. El-Naqeeb and Abdelwahed^7^ focused on the differences in punching shear behavior between thick and thin plates, particularly under eccentric loading conditions. Their numerical study developed and validated a model to examine the punching shear behavior of full-scale eccentrically loaded footings. The model accurately predicted the behavior of footings under various eccentricity ratios, revealing the significant influence of load eccentricity on punching shear strength and shear crack distribution. The study also highlighted the importance of column perimeter to depth ratio and shear span to depth ratio in determining footing behavior. The prEC2 approach for predicting shear strength was found to be inaccurate for eccentric loading cases, prompting the development of a new, more accurate equation.

In the context of ultra-high-performance concrete (UHPC) post-tensioned flat slabs, research by Afifi et al.^8^ explored the impact of column dimensions and punching reinforcement on punching capacity. This study involved testing five slabs, revealing that ACI-318 predictions were more accurate compared to EC2. Additionally, the presence of punching reinforcement increased the crack angle and enhanced ductility and stiffness. Similarly, Ramadan et al.^9^ investigated the behavior of high-strength concrete (HSC) and UHPC post-tensioned flat slabs, proposing correction factors for ACI-318 and EC2 based on FEM model validation and parametric studies. Their research demonstrated the potential of machine learning techniques to predict correction factors with high accuracy. Elsheshtawy et al.^10^ evaluated the influence of post-tensioning force distribution and strand layout on the punching shear behavior of flat slabs. Their findings indicated that increased pre-stressing force and a banded strand layout significantly enhanced punching shear capacity and ductility. Furthermore, a comparison with different design codes showed varying degrees of conservatism and accuracy. Additionally, Ors et al.^11^ presented a study on evaluating the lateral subgrade reaction of soil using horizontal pile load tests. Their research developed a correlation between the coefficient of variation of lateral subgrade reaction and the elastic modulus of soil, providing insights into the disparity between experimental and recommended values in codes. Ebid and Deifalla^12^ developed three AI based models to predict the punching capacity of lightweight concrete slabs. They considered column dimensions, slab thickness, punching shear reinforcement, concrete density and compressive strength.

**Fig. 1:**
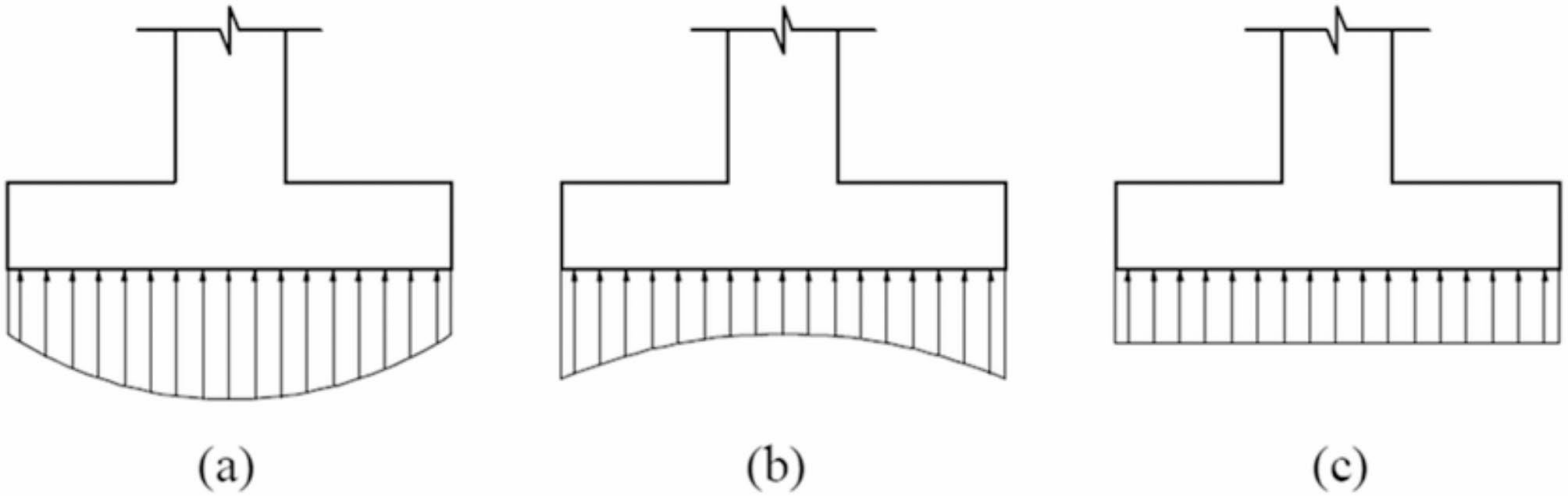
Stress distribution below footings (**a**) for granular soil, (**b**) for cohesive soil and (**c**) Idealized distribution.

The contact pressure distribution beneath RC footings is affected by both soil type and footing stiffness changes post-cracking. As shown in Fig. 1, Talesnick^13^ introduced the Null Soil Pressure System to address the complexities of soil pressure measurement within a soil mass. The study embedded and tested null pressure sensors in various uniformly graded soils, finding that the response consistently exceeded actual soil pressure by 4%±3%, independent of soil type, particle size, stiffness, and stress history. This method showed no hysteresis between loading and unloading, providing a reliable measure of soil pressure within a soil mass. Fouda et al.^14^ utilized ABAQUS 6.14 to perform numerical simulations and validations for full-scale square RC footings on sand soil, focusing on the effect of concrete damage plasticity on contact stress distribution. The study found that as footing stiffness decreases during loading, the percentage of contact stresses at the edges relative to those under the column center also decreases. A parametric study revealed that increasing the reinforcement ratio from 0.15 to 0.30% significantly affects the failure load and the contact stress distribution before and after cracking. These findings challenge the theoretical assumptions of contact stress distribution under square footings. Reliable measurement of soil pressure within a particulate media has been a longstanding challenge in soil mechanics and soil-structure interaction.

The bearing capacity and settlement behavior of footings are critical considerations in geotechnical engineering, especially when these footings are reinforced with geosynthetics like geogrids. Research in this field has focused on understanding how different reinforcement configurations affect the performance of footings under various soil conditions. Kumar and Saran^15^ conducted a comprehensive study involving 74 tests on closely spaced strip and square footings on geogrid-reinforced sand. Their research evaluated the effects of footing spacing, reinforcement size, and the use of continuous versus discontinuous reinforcement layers on bearing capacity and tilt. They found that while interference effects on bearing capacity and settlement were minimal for closely spaced square footings compared to isolated footings, continuous reinforcement layers significantly improved the tilt of adjacent footings. Additionally, substantial improvements in bearing capacity, settlement, and tilt were observed for closely spaced strip footings with continuous reinforcement layers. Biswas et al.^16^ examined geogrid-reinforced foundation systems supported on clay subgrades of varying strengths. Their model tests on circular footings highlighted that planar geogrid reinforcement at the sand-clay interface substantially improved foundation performance, particularly in terms of bearing capacity, which saw up to a 5.6-fold increase for very soft clay subgrades. The effectiveness of reinforcement was closely tied to the thickness of the sand layer and the strength of the subgrade. Satvati et al.^17^ investigated the bearing capacity of shallow footings reinforced with braid and geogrid near soil slopes. Their study found that cylindrical reinforcement (braid) performed better than planar reinforcement (geogrid) in enhancing bearing capacity and reducing settlement. The inclusion of geosynthetics helped distribute applied loads and mitigate stress concentrations, resulting in improved footing performance.

**Fig. 2:**
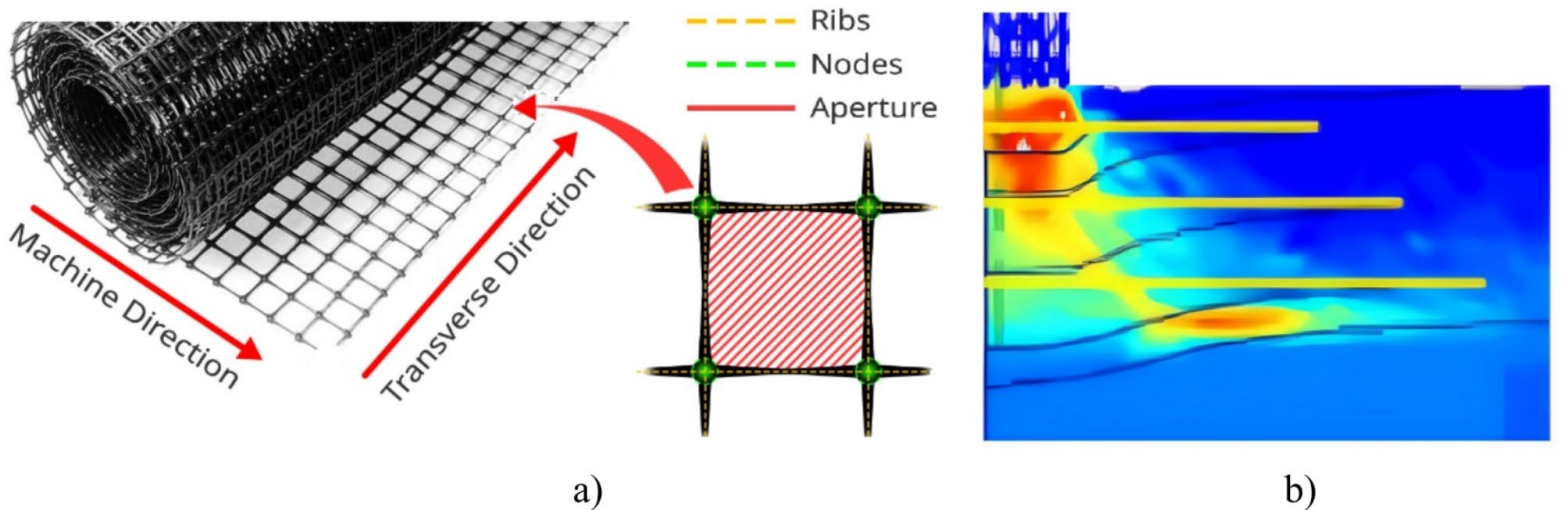
(**a**) Bi-directional geogrid, (**b**) stress distribution through geogrid.

Further extending this line of inquiry, Ahmad et al.^18^ investigated the bearing capacity of strip footings improved by wraparound geogrid sheets through both experimental and numerical analyses. They assessed the effects of fully folded geogrid layers on fine sand, noting significant improvements in bearing capacity and reductions in settlement. The study proposed a modified expression for bearing capacity that closely aligned with experimental results, demonstrating a low error rate of approximately 7%. Ebid et al.^19^ explored the load-settlement response of strip foundations with different arrangements of geogrid inclusions in fine sand as illustrated in Fig. 2. Their experiments revealed that foundations reinforced with folded geogrid layers exhibited stiffer behavior and supported higher loads with lower settlements compared to those with planar geogrid layers. Key parameters such as the number, thickness, and placement of folded geogrid layers were critical in enhancing the performance. The study recommended specific embedment depths and vertical spacing for optimal reinforcement configurations. Hussain et al.^20^ examined the use of geogrid reinforcement to improve the bearing capacity and stability of square foundations on sandy soil, typical of Karbala. The study investigated the effects of geogrid depth, size, and number of layers on bearing capacity and soil settling. Results indicated that decreasing the depth ratio of geogrid layers significantly increased bearing capacity and settlement reduction. The optimal configuration was found to be a depth ratio of u/B = 0.5, a size ratio of b/B = 4.5, and three geogrid layers, which provided the highest bearing capacity and settlement reduction. These experimental results were verified against theoretically developed models, showing reasonable variation and confirming the high accuracy of the experimental findings.

**Fig. 3:**
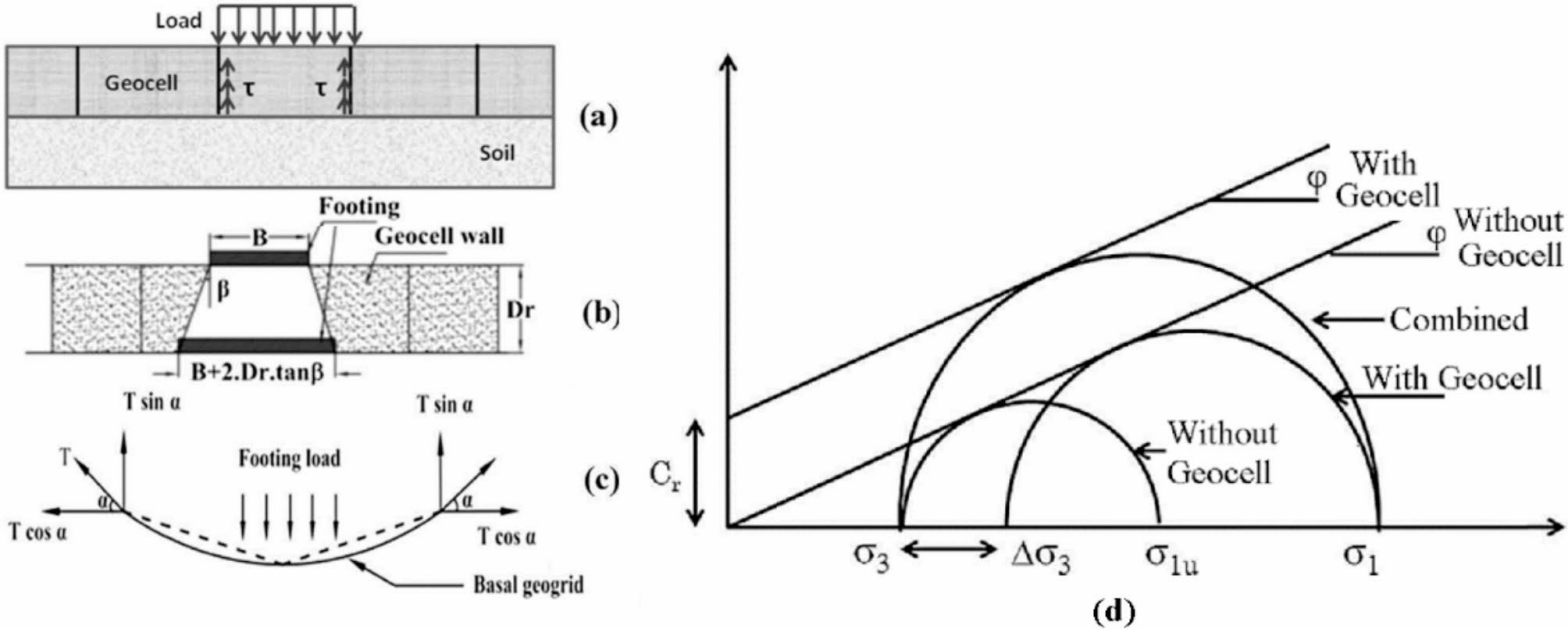
Load transfer mechanisms and failure envelope of confined soil in geocell. (**a**) Lateral resistance, (**b**) vertical dissipation, (**c**) membrane action and (**d**) failure envelop.

The influence of geocell confinement on the mechanical properties of granular soils was investigated by Rajagopal, Krishnaswamy, and Latha^21^. Through extensive triaxial compression tests on soils encased in single and multiple geocells made from various geotextiles, the study demonstrated that geocell confinement substantially increases the apparent cohesive strength and stiffness of granular soils. This enhancement is dependent on the geosynthetic material properties, with a simple methodology proposed to estimate the cohesive strength based on the geocell’s geometric and material properties (see Fig. 3). The application of geocell foundations has shown significant promise in enhancing the stability and load-bearing capacity of structures built on soft or problematic soils. One notable study by Hegde and Sitharam^22^ discusses the design and construction of a geocell foundation to support a 3-meter-high embankment over soft settled red mud, a byproduct of the aluminum industry. This research emphasizes the geotechnical challenges of the site and the effectiveness of geocell and geogrid combinations in stabilizing the embankment base. The study’s experimental investigations and subsequent analytical model revealed that using both geocell and geogrid provides superior load-carrying capacity compared to geocell alone. The practical implementation of this foundation system in Lanjigharh, Orissa, demonstrated its durability, as the constructed embankment withstood multiple monsoon seasons without significant damage.

Further advancements in geocell modeling techniques have been explored by Hegde^23^, who addressed the challenges of accurately representing geocells’ complex honeycomb structures in numerical simulations. They developed a more realistic 3-dimensional model using FLAC3D, which incorporated geogrid structural elements and interface elements. Their study showed that geocells significantly enhance load distribution and performance of reinforced foundation beds. The validated numerical model, which closely matched experimental results, indicated that factors such as geocell modulus, height, pocket size, and surface texture critically influence the reinforced bed’s performance. Recent research by Demirdöğen, Gürbüz, and Yünkül^24^ examined the performance of eccentrically loaded strip footings on geocell-reinforced sand. Their laboratory model tests evaluated pressure-settlement responses, surface displacement profiles, failure mechanisms, and ultimate bearing capacity, considering factors such as load eccentricity, geocell height, material stiffness, and soil relative density. The findings indicated that geocell reinforcement significantly improves bearing capacity, with up to a 6.5-fold increase compared to unreinforced soils. The study also proposed a design chart for predicting failure modes and a new method for evaluating ultimate bearing capacity. It was observed that increasing soil relative density and geocell material stiffness enhances bearing capacity, although geocell height had a negligible impact. These studies collectively underscore the critical role of geocell foundations in improving the structural performance of footings on challenging soils. They highlight the need for continued research and development to optimize geocell design and application, ensuring greater stability and longevity of constructed embankments and foundations.

Finally, various reinforcement configurations, such as vertical encasement and horizontal geogrid strips, have been proposed and tested for their effectiveness in different conditions. Hasan and Samadhiya^25^ conducted laboratory model tests and numerical simulations on granular piles reinforced with geosynthetics in soft clay soils, investigating configurations including vertical encasement, horizontal strips, and combined vertical-horizontal reinforcement. Their findings reveal a marked increase in both ultimate load intensity and stiffness of the treated ground, emphasizing the effectiveness of geosynthetic inclusion in stabilizing soft clay foundations under load.

Further research by Ghazavi and Afshar^26^ highlights the role of stone columns in enhancing the bearing capacity of soils with low strength. Their study explores the impact of various diameters and reinforcement configurations on stone column performance, particularly focusing on vertically encased stone columns (VESC). They observed that geosynthetic reinforcement notably improves load-bearing capacity and reduces lateral bulging, with larger columns and increased reinforcement strength further enhancing the bearing capacity. Finite element analysis supplemented their physical tests, demonstrating the potential for expanding the reinforcement application to larger-scale structures.

In addition, Hasan and Samadhiya^27^ studied granular piles reinforced with horizontal geogrid strips, investigating both floating and end-bearing configurations in very soft clay. Their experimental and numerical results confirm that incorporating horizontal geogrid reinforcement significantly improves the load intensity and minimizes bulging in granular piles, with load-bearing capacities increasing by up to 442% for treated ground compared to untreated soil.

## Objective

As outlined in the introduction, the use of geocells and geogrids in geotechnical applications has a positive and quantifiable impact on foundation behavior, enhancing load-bearing capacity, reducing settlement, and promoting overall stability in various soil conditions. However, there is a gap in the research regarding the effect of geocell and geogrid on the punching capacity of shallow foundations. Therefore, the primary objective of this study is to experimentally investigate the impact of increasing the stiffness of the soil beneath isolated footing using replacement, geocell or geogrid on its punching capacity.

## Methodology

The objective of this research is achieved through a series of experimental tests designed to investigate the effect of increasing soil stiffness beneath footings using geocells and geogrids, as well as testing the impact of various soil types. During the loading of the tested footing specimens, measurements were taken for load, settlement, strains in the reinforcing steel, and pressure at the soil/footing interface. A comprehensive analysis was then performed to determine the influence of each parameter on the punching shear behavior of the reinforced concrete footings, focusing on failure mechanisms, load-settlement response, stress distribution at the soil/footing interface, and the modulus of subgrade reaction.

### The experimental program

**Fig. 4:**
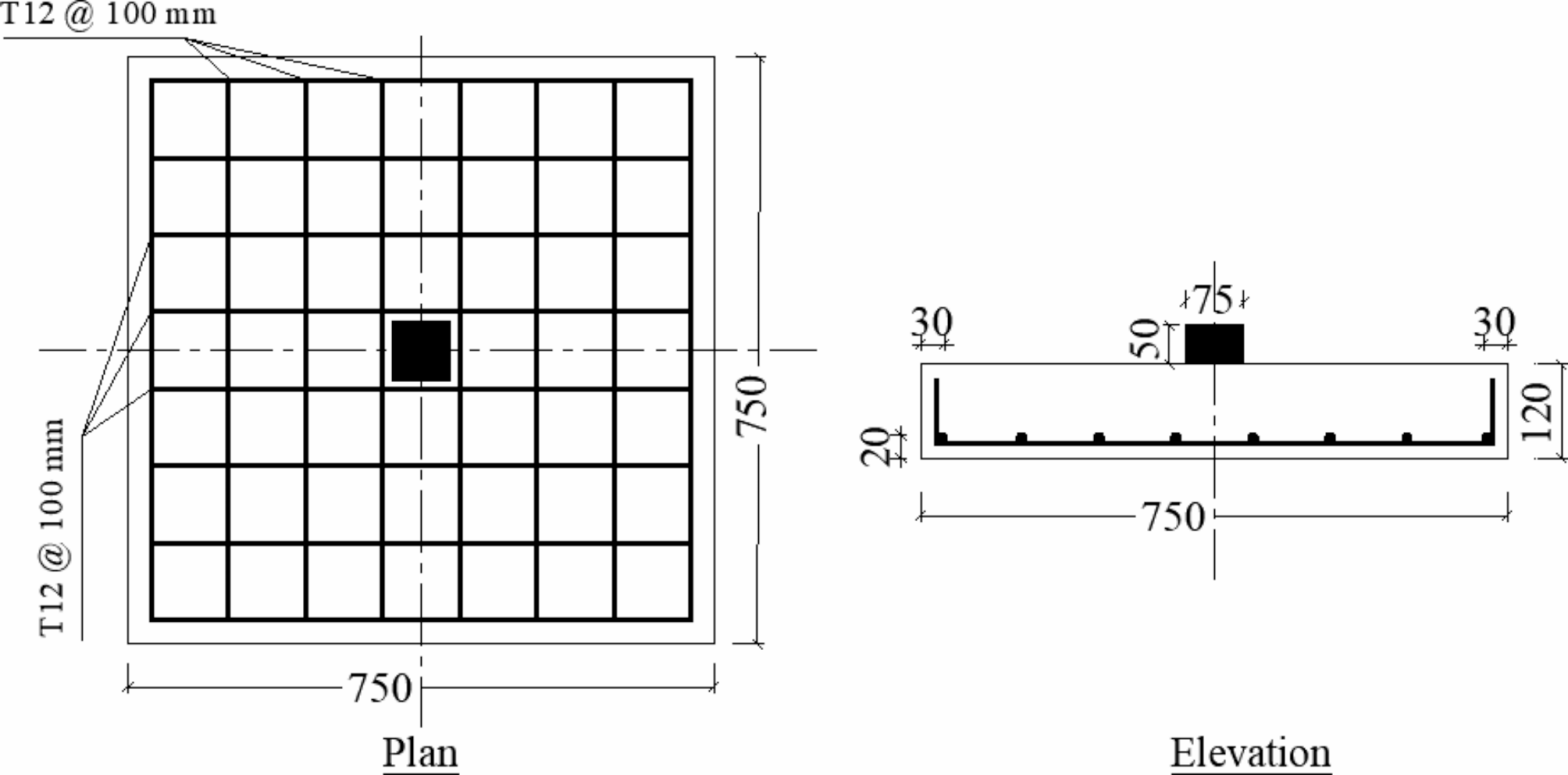
Typical footing configurations for all tested footing specimens (all dimensions are in mm).

**Fig. 5:**
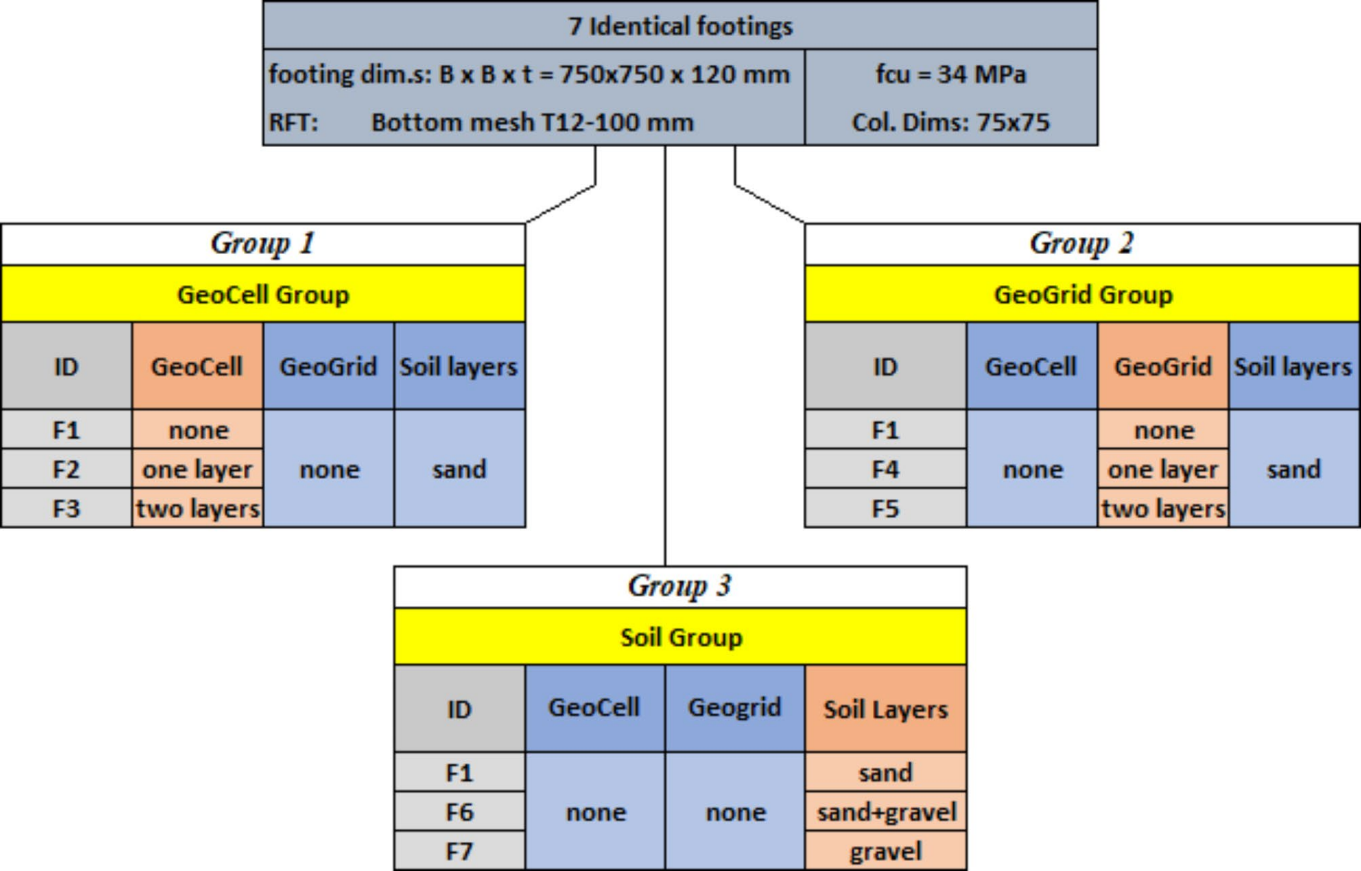
Experimental matrix.

The experimental program conducted in this study involved seven identical isolated footings (750 × 750 × 120 mm) placed on various soil types and subjected to a monotonic centric load until failure. A steel plate (75 × 75 mm, 50 mm thick) was used to simulate the column at the center of each footing. Figure 4 depicts the typical configurations of the tested footings. The first footing, serving as the control, was placed on dense sand without the use of geocell or geogrid, while the remaining six footing specimens differed in the number of geocell / geogrid layers and type of soil beneath them. The experimental matrix was divided into three groups based on the tested parameter. Group 1 consisted of F1, F2, and F3, constructed on sand soil reinforced with different numbers of geocell layers (1200 × 1200 × 140 mm) centered beneath the footings. Group 2 comprised F1, F4, and F5, featuring varying numbers of geogrid layers (1250 × 1250 mm). Finally, Group 3 involved varying the soil type beneath the footings, with dense sand for the control specimen F1, a 1:1 mixture of sand and crushed stone for F6, and crushed stone alone for F7. Figure 5 organizes the tested footing specimens into groups according to the tested parameters.

### Material testing and characteristics

#### Concrete

All footings were cast using the same concrete mix. Three cubes 150 × 150 × 150 mm were cast with the footings. Both footings and cubes were cured with clean water for 28 days, and then the cubes were tested in compression. The recorded strengths were 32.8, 33.6, 35.4 MPa with average value of 34.0 MPa. Ordinary Portland cement (CEM-I) with strength of 42.5 MPa were used in this mix. The coarse aggregates were crushed dolomite with maximum nominal size of 10 mm. the measured slump was 135 mm. The concrete mix proportions are illustrated in Table 1.

**Table 1:** Concrete mix proportions per 1.0 m^3^.

Cement (kg)	Sand (kg)	Aggregate (kg)	Water (kg)
350	727	1146	205

#### Reinforcement rebars

All footings were reinforced with lower mesh of 8T12 in both directions. The used rebars were high strength ST (50/70) with minimum yield strength of 500 MPa as manufacture certificate.

#### Soils

**Fig. 6:**
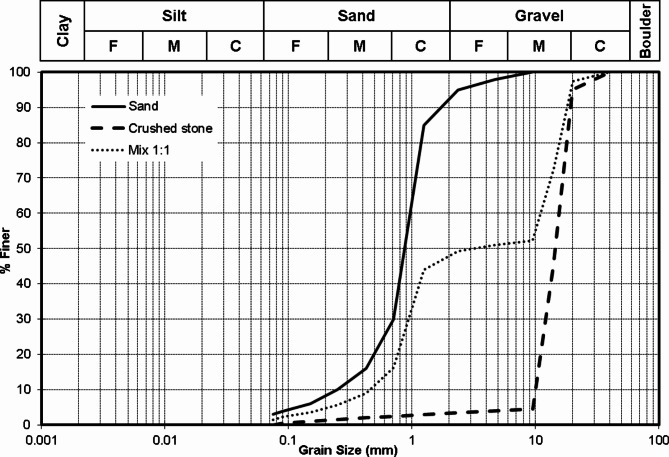
Grain size distribution of used soil types.

**Fig. 7:**
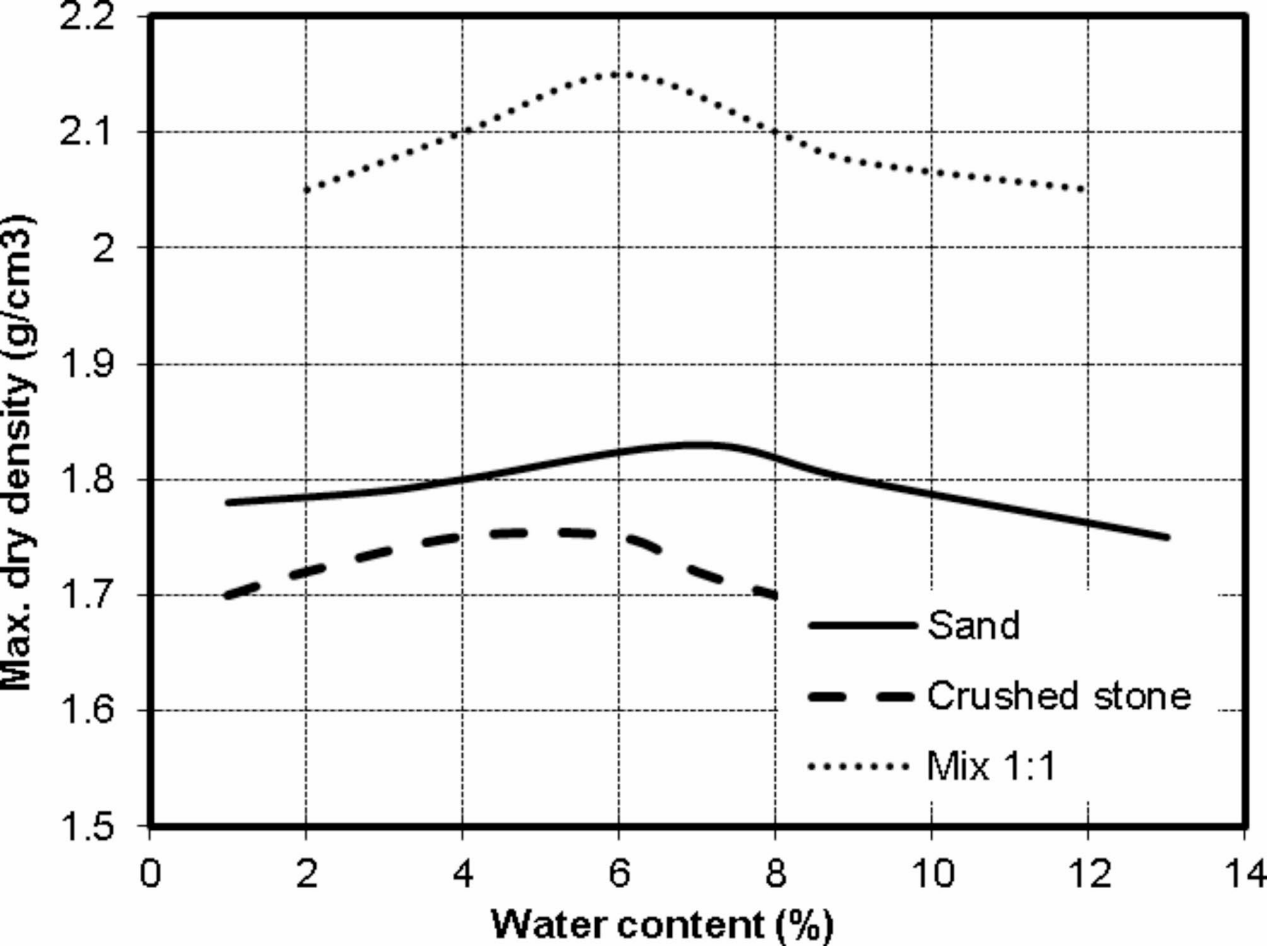
Modified proctor test results of used soil types.

**Fig. 8:**
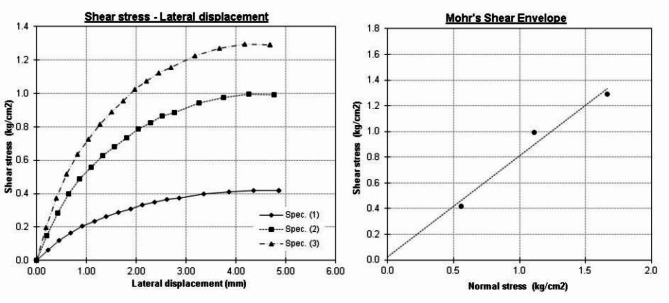
Direct shear test results of used soil.

Three types of soil were used in this study, siliceous sand, crushed limestone and a mix of them (1:1). Figure 6 presents the grain size analysis of the three types. It could be noted that the sand is well grade and the crushed limestone has a uniform size of (10 to 20 mm). Accordingly, the (1:1) mixture has a gap in grain sizes between 2 and 10 mm (medium and fine gravel). The presented modified Proctor test results in Fig. 7 showed that values of maximum dry density and optimum water content are (1.83 g/cm^3^, 7%), (1.77 g/cm^3^, 5%) and (2.15 g/cm^3^, 6%) for sand, crushed stone and (1:1) mix respectively. Finally, direct shear test results indicated that the angle of internal friction of the sand was 38.0 degree while the cohesion is almost zero as shown in Fig. 8. All soil tests were conducted in NECB Labs according to the Egyptian Code of Practice for soil mechanics—Part 2: Lab tests “ECP202/2”.

#### Geocell

The used geocell layers were provided by LSF GEOCELL EGYPT Co. The cell was perforated and with dimensions of 210 × 250 mm, height of 140 mm and fabric thickness of 1.4 mm (ASTM D- 5199). The technical specifications as per the datasheet were:

- Material composition: UV Resistant Polyethylene( HDPE).
- Material Density: 0.95 kg/m^3^ (ASTM D- 1505).
- Tensile Strength: 14.0 kN/m (EN ISO 10319).
- Seam Peel Strength: 1.0 kN (EN ISO 10319).
- Welding Type: Ultrasonic.

#### Geogrid

The used geogrid layers were biaxial geogrid BX1500 manufactured by Tensar Co. The technical specifications as per the datasheet were:

- Material composition: Polypropylene.
- Aperture Dimensions: 25 × 25 mm.
- Minimum Rib Thickness 1.78 mm.
- Tensile Strength, 2% Strain 8.5 kN/m (BS EN ISO 10319:2015).
- Tensile Strength, 5% Strain 17.5 kN/m (BS EN ISO 10319:2015).
- Ultimate Tensile Strength 27.0 kN/m (BS EN ISO 10319:2015).

### Instrumentations

**Fig. 9:**
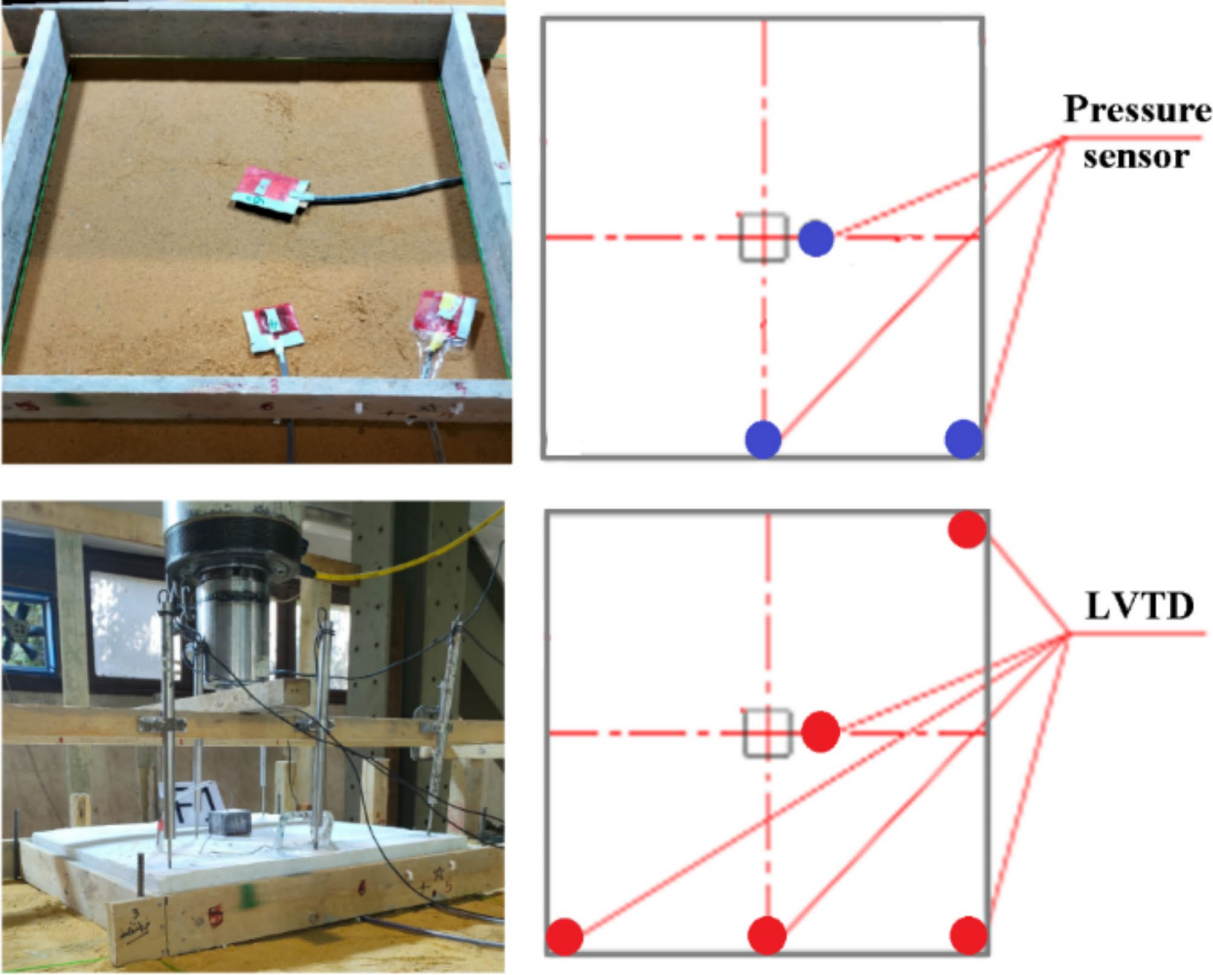
Locations of pressure sensors and LVTD’s.

All tested footings were equipped by strain gauge at the center of the middle rebar as shown in Fig. 9 (at the location of maximum bending moment) to measure the stresses in the rebars and assure that the failure is not flexural. Besides that, Five LVTD’s were used to measure the settlement at the center, mid edge and corner of the footing. In addition, three pressure sensors were used below the footing to measure the contact pressure at the center, mid edge and corner of the footing as shown in Fig. 9. For the second and third footings, additional three pressure sensors were placed below the geocell layer & the geogrid layer in the same locations of the upper ones. Finally, a load cell was used to measure the applied load during the test. All instrumentations readings were automatically recorded by data acquisition system.

### Testing setup and stages of construction

**Fig. 10:**
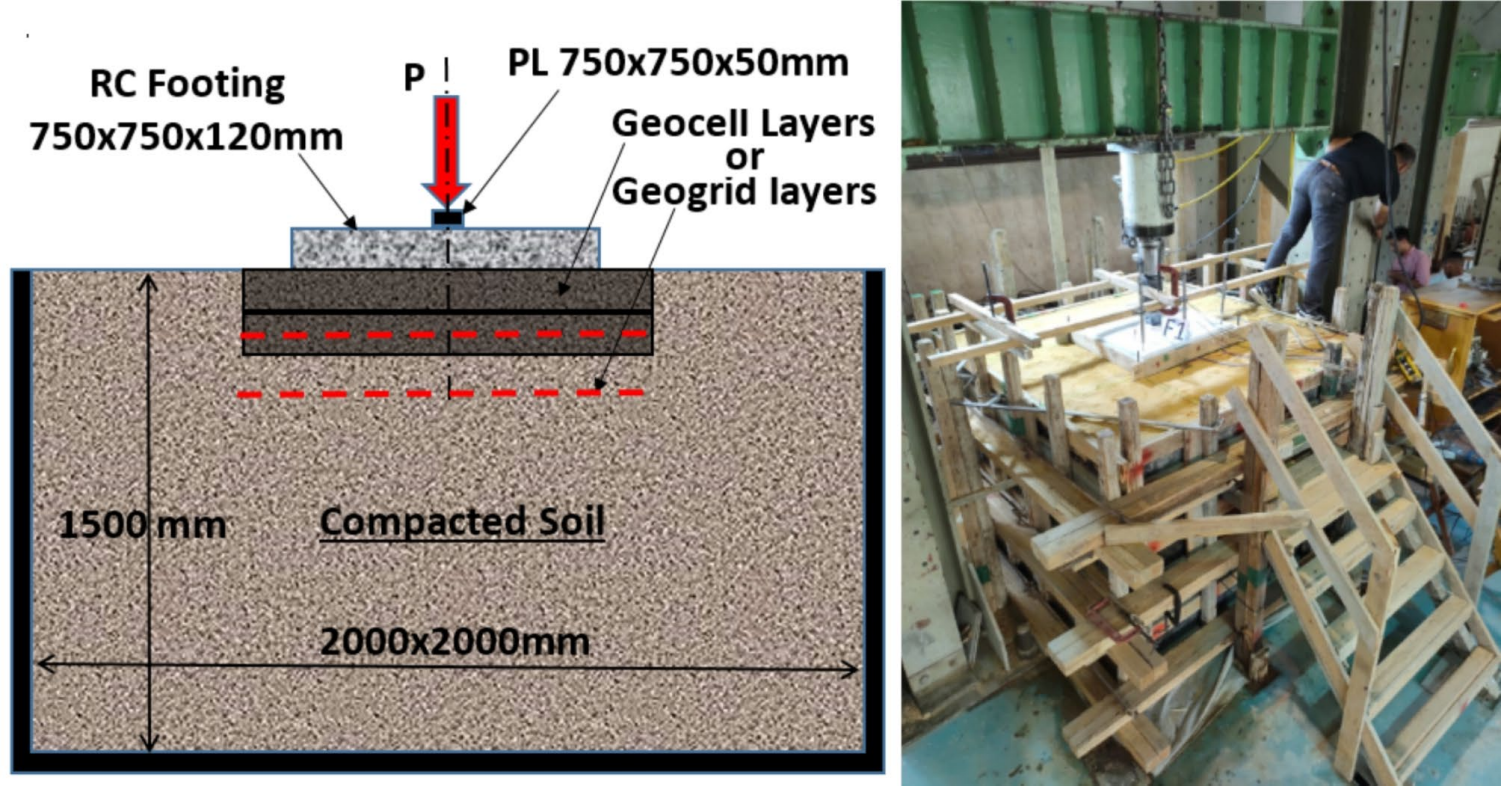
Test setup.

The loading tests were conducted in the Concrete Laboratory of the Faculty of Engineering, Ain Shams University. The experimental setup is illustrated in Fig. 10, where a sandbox measuring 2.0 × 2.0 × 1.5 m was positioned beneath the testing frame. The box was incrementally filled with sand and compacted in 15 cm layers using a plate compactor, achieving 95% of the dry density as determined by the modified Proctor test. The first footing (F1) was placed centrally below a 1000 kN capacity hydraulic jack, aligned over pre-positioned pressure sensors. Both the loading plate and linear variable differential transformers (LVDTs) were secured in position, and the footing was gradually loaded at a rate of 5.0 tons per minute until failure (indicated by a reduction in applied load) was observed.

**Fig. 11:**
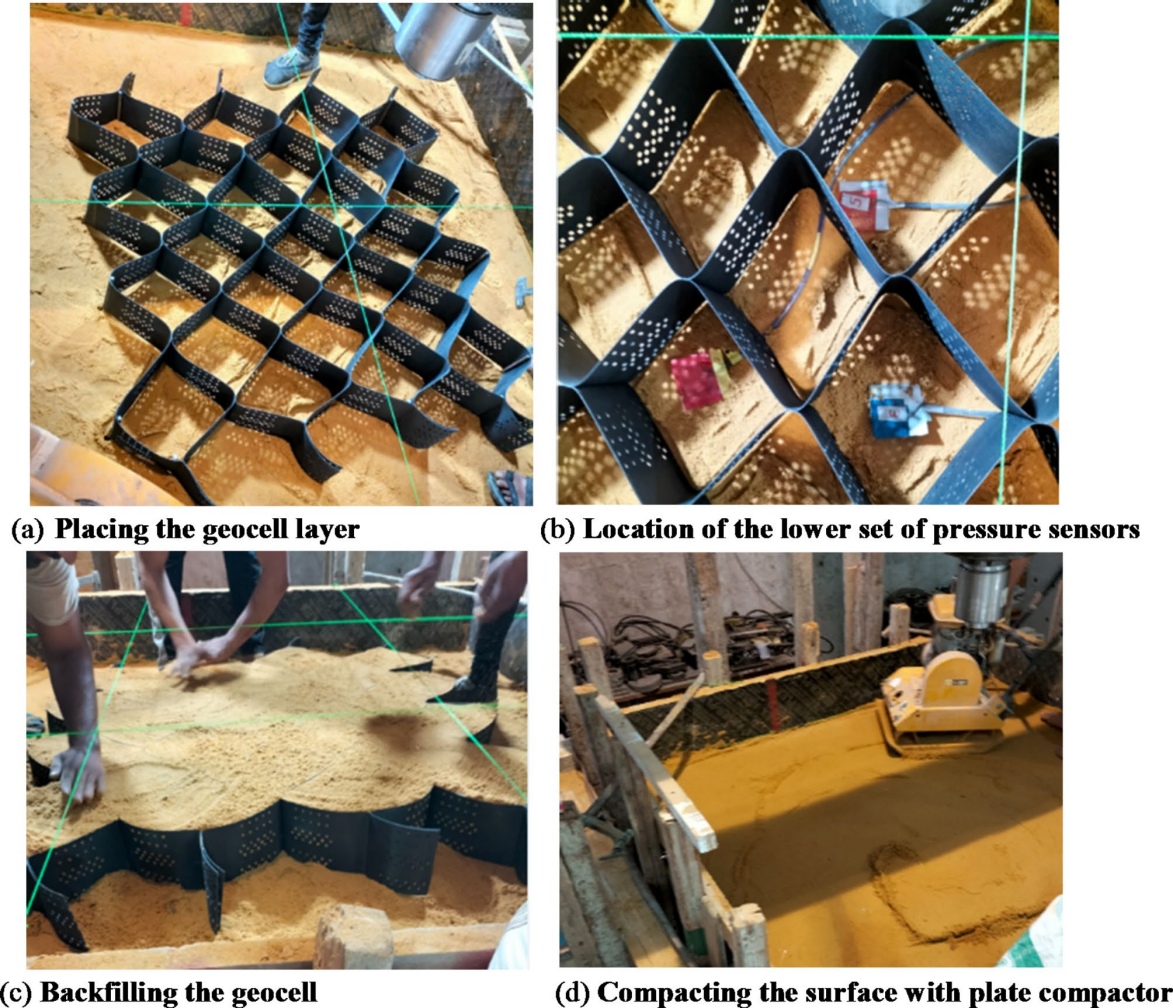
Preparing the geocell below samples (F2, F3).

Following removal of the first footing, the sandbox was emptied, refilled, and re-compacted using the same procedure. Before adding the final layer of sand, a lower set of pressure sensors was installed, and a geocell layer was positioned and secured with steel rods at the corners, then filled and compacted as shown in Fig. 11. The second footing (F2) was then placed and tested similarly to the first.

After the second footing test, the box was emptied and refilled to a height of 1220 mm. A set of lower pressure sensors and geocell layers were installed, filled, and compacted, followed by an upper geocell layer installed and compacted using the same method. The third footing (F3) was placed on this prepared surface, with the loading plate and LVDTs secured, and loaded to failure.

**Fig. 12:**
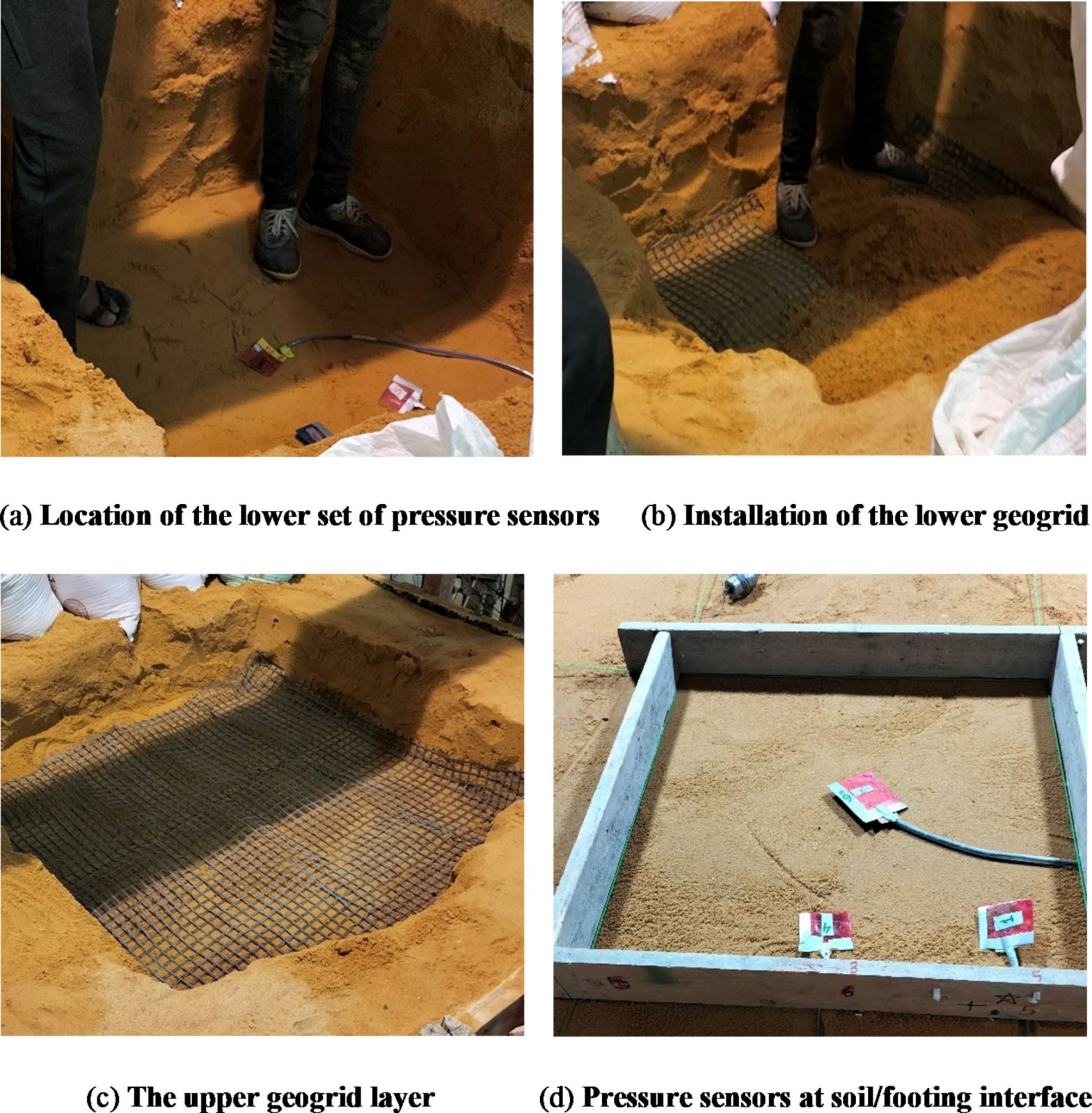
Preparing the geogrid below samples (F4, F5).

The sandbox was subsequently emptied and refilled to 1250 mm, following the same compaction procedure. The lower set of pressure sensors was installed, followed by the placement and compaction of a geogrid layer as shown in Fig. 12. The fourth footing (F4) was then positioned and tested under the same conditions as the previous footings. The box was then emptied and refilled up to 1000 mm, where a lower set of pressure sensors and a lower geogrid layer were installed and compacted to 1250 mm. An upper geogrid layer was then added and compacted in a similar manner, after which the fifth footing (F5) was tested up to failure.

**Fig. 13:**
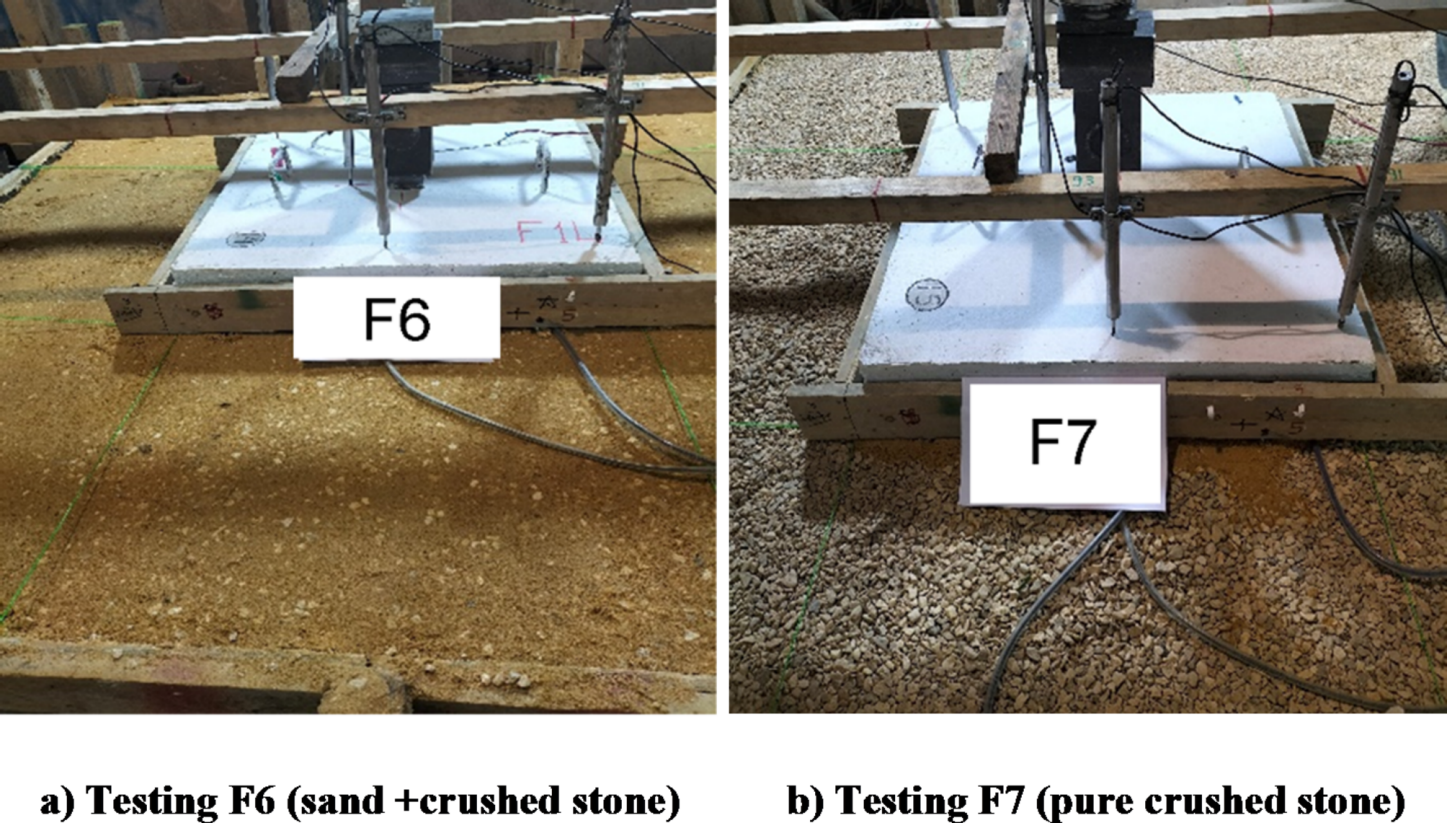
Preparing different soils below samples (F6, F7).

Following the removal of the fifth footing, the sandbox was emptied, refilled with a second type of soil (a 1:1 mix of sand and crushed stone), and compacted in 15 cm layers. The sixth footing (F6) was placed on the final surface, with pressure sensors in place as shown in Fig. 13, and tested to failure. Subsequently, the box was emptied again, refilled with a third type of soil (pure crushed stone), and compacted as before. The seventh footing (F7) was positioned over pre-located pressure sensors as depicted in Fig. 13, and tested to failure under identical conditions.

## Results and dissection

### Failure mode and crack pattern

**Fig. 14:**
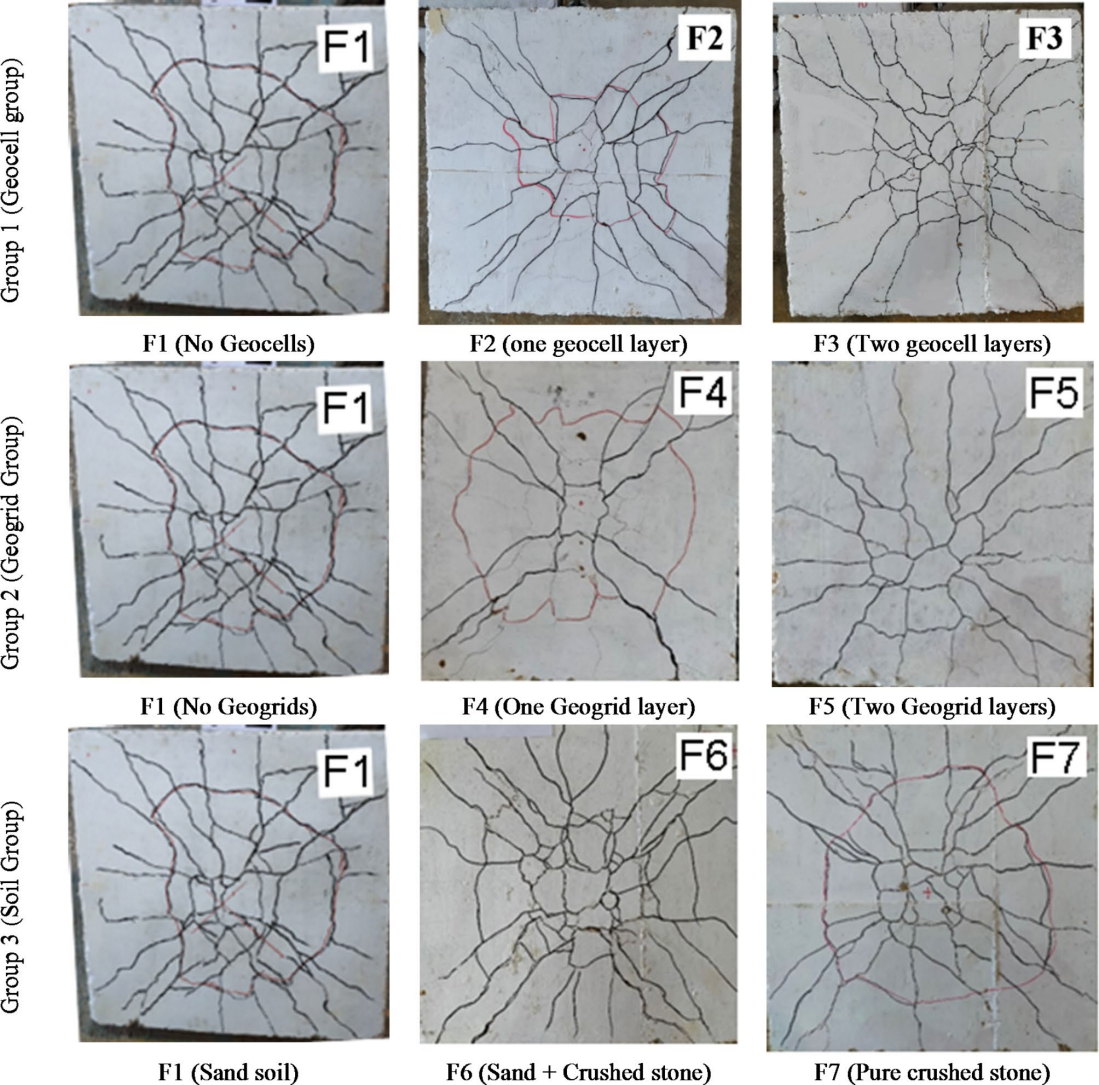
Crack pattern at bottom footing surface at failure.

The observed brittle and sudden failure in all samples indicated a pure punching failure. Figure 14 presents the crack patterns of the samples. It shows a typical punching failure patterns with radial cracks from the center to the edges, and almost circular crack that defines the base of the punching cone. Moreover, it illustrates that the diameter of punching cone base of F1 is about 500 mm, which is larger than those of F2 and F3 (about 350 to 400 mm).

In addition, the recorded strains in main reinforcement rebars were less than the yielding strain (2500 µ-strain) as shown in Table 2. These values assure that the failure mode is not flexural failure.

### Load-settlement behavior

**Fig. 15:**
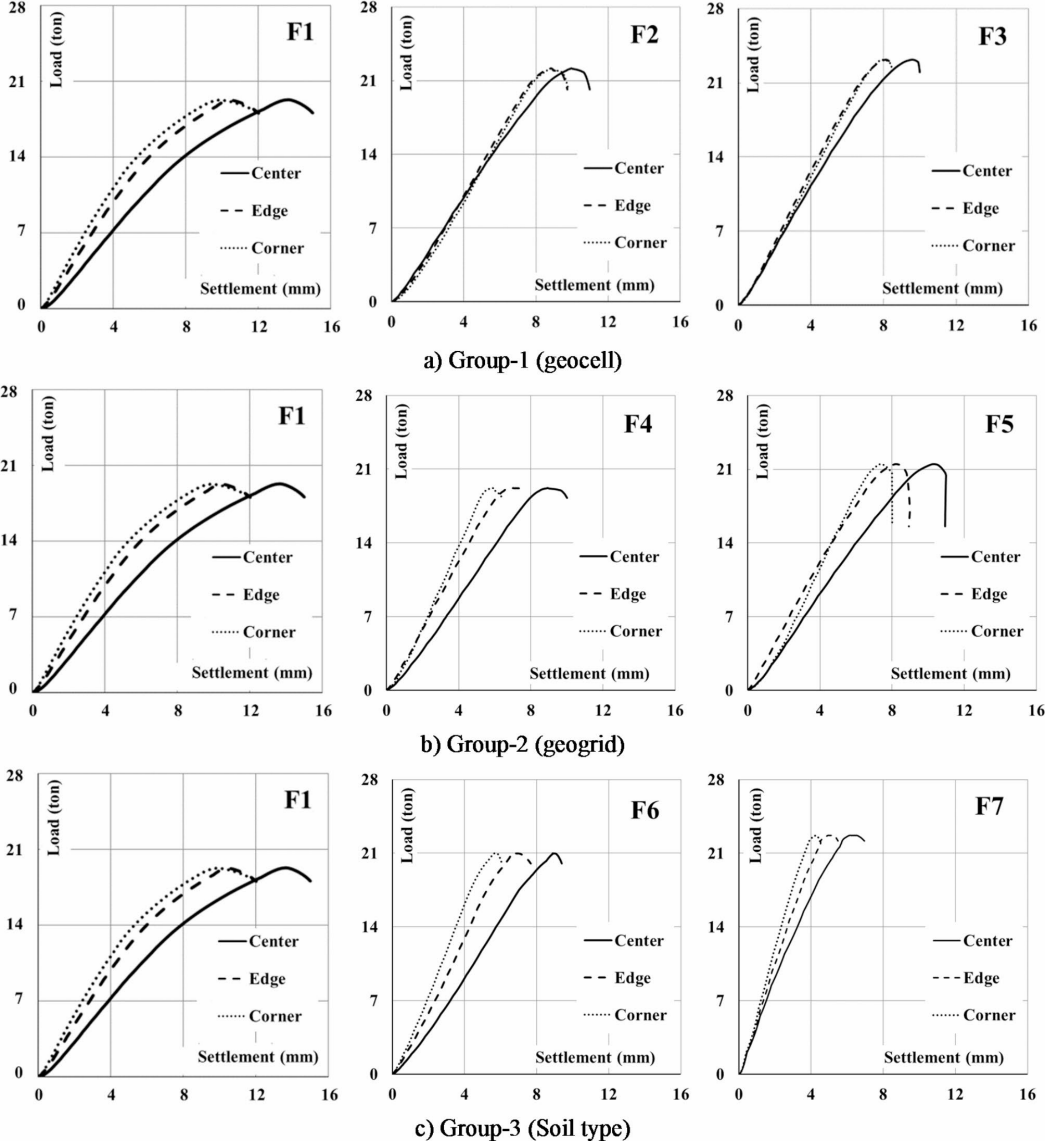
(Load- settlement) curves just below the footing at its center, mid-edge and corner.

Settlement values were continually recorded at different points and levels during the test. At center, corner and mid edges of the footing, just below the footing and below soil reinforcement layers as summarized in Table 2. Figure 15 shows the (load-settlement) carvers just below the footings. It illustrates the brittle and sudden failure behavior of punching shear failure. Analyzing these values shows that the relative settlement at the edge and corner with respect to the maximum settlement under the center of the footing are (77%, 72%) for (F1), (88.9%, 89.4%) for (F2) ,(84.6%, 83.4%) for (F3), (77.6%, 66.3%) for (F4), (79.7%,71.3%) for (F5), (74.6%,64%) for (F6) and (80.4%,72%) for (F7). The maximum settlement values at the failure are listed in Table 2.

**Table 2:** Summary for the recorded results.

Footing	Pult (ton)	Δ ult (mm)			σ _max_ below footing (t/m^2^)			σ _max_ below Geocell (t/m^2^)			σ _max_ below Geogrid (t/m^2^)			ε max (µ-strain)
		Center	Edge	Corner	Center	Edge	Corner	Center	Edge	Corner	Center	Edge	Corner	
F1	19.3	13.7	10.5	9.9	44	26	18	–	–	–	–	–	–	1974
F2	22.2	10.1	8.9	8.9	59	22	4	44	35	27	–	–	–	2177
F3	23.2	9.3	8.1	8.0	61	21	4	46	36	23	–	–	–	2350
F4	19.2	10.3	8.23	7.4	46	25	14	–	–	–	46	24	17	2138
F5	21.5	10.5	9.5	8.2	57	24	6	–	–	–	47	34	19	1760
F6	21.0	9.2	9.0	8.2	53	24	8	–	–	–	–	–	–	1631
F7	22.7	6.8	5.6	4.9	62	22	2	–	–	–	–	–	–	2306

### Influence of different parameters on the load-settlement response

**Fig. 16:**
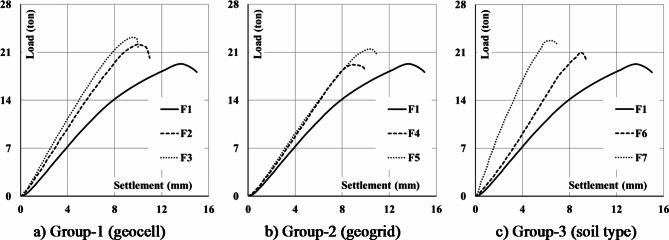
The impact of the considered parameters on the load-center settlement response.

A comparative analysis was conducted within a consistent group of footings to evaluate the effects of incorporating geocell and geogrid layers, as well as different soil types, across groups 1, 2, and 3. The assessment of ultimate loads and corresponding settlements at the center of the footings in-group 1 demonstrated an increase in punching capacity by 14.9% (22.19/19.3) and 20.1% (23.18/19.3) with the application of one and two layers of geocell, respectively. Concurrently, settlements at the center decreased by 72.6% (9.95/13.7) and 70% (9.59/13.7) for one and two layers of geocell, respectively (Fig. 16a). The load-settlement relationship for F1, F4, and F5 is illustrated in Fig. 16b. A comparison of the ultimate loads and corresponding settlements at the center indicated that the punching capacity of the footing increased by 0% (19.2/19.3) and 11% (21.5/19.3) with the inclusion of one and two layers of geogrid, respectively. Additionally, the observed reductions in settlement at the center were 65% (8.93/13.7) and 75% (10.31/13.7) for footings with one and two layers of geogrid, respectively. The load-settlement relationship is presented in Fig. 16c for F1, F6, and F7. Comparing the ultimate loads and corresponding settlements at the center revealed an increase in punching capacity by 8% (21.0/19.3) and 17% (22.7/19.3) when using crushed stone and sand mixture for F6 and pure crushed stone for F7. The observed reductions in settlement at the center were 64.8% (8.88/13.7) and 45.4% (6.23/13.7) for F6 and F7, respectively.

### Contact pressure distribution

**Fig. 17:**
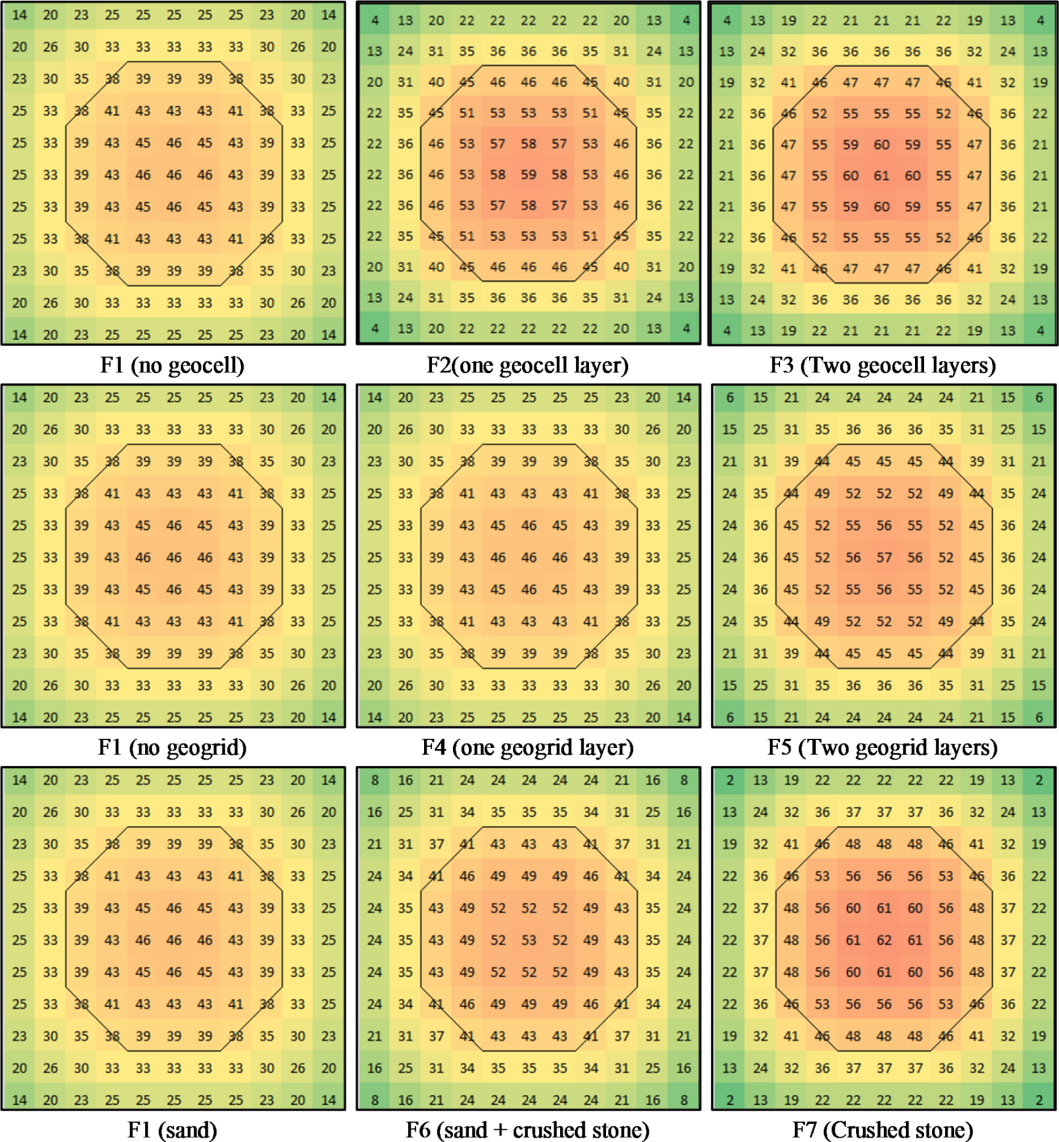
Contact pressure contours (t/m^2^) just below footing at failure.

**Fig. 18:**
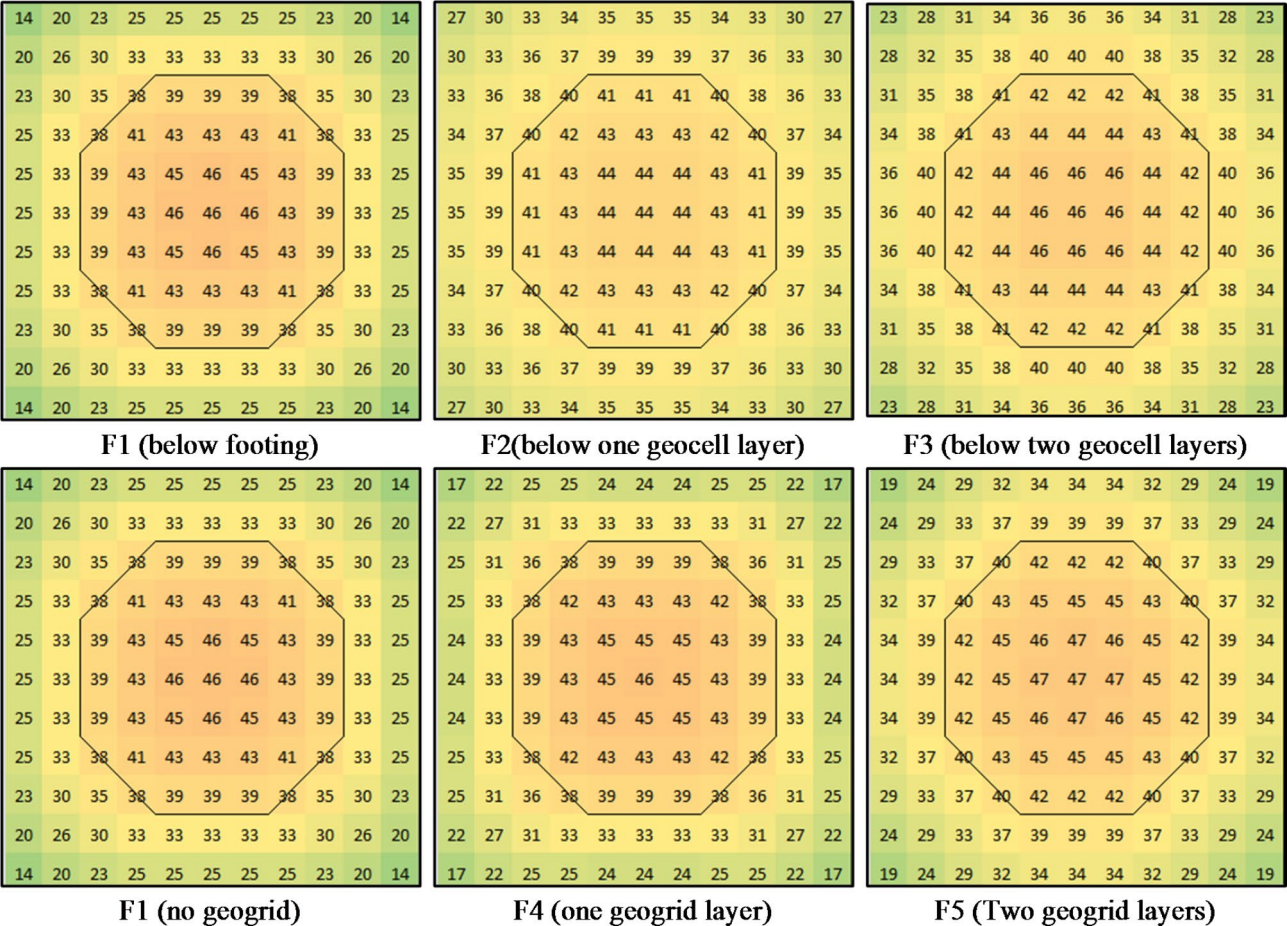
Contact pressure contours (t/m^2^) below the reinforced soil layer at failure.

Contact pressure was measured at three points beneath the footing: the center, mid-edge, and corner. These measurements were used to generate a contact pressure contour through interpolation and symmetry. The resulting contours for contact pressure just below the footing are presented in Fig. 17, while the contact pressure distribution below the geocell/geogrid layers are presented in Fig. 18. The contours indicate that the relative contact pressure at the edge and corner to the center for groupe-1 footings were 54% (25/46) and 30% (14/46) for F1, 37% (22/59) and 7% (4/59) for F2, and 34% (21/61) and 6% (4/61) for F3. This suggests that using geocell layers concentrates the stress below load by approximately 12% (59/46) for one layer and 26% (61/46) for two layers. For the contact pressure below the geocell layers, Fig. 18 shows an almost uniform distribution with an average value of 40.5 t/m² ± 8.6% for F2 and 43.5 t/m² ± 5.7% for F3. This enhancement in stress distribution indicated that using geocell layers increases the stiffness of the soil by confining. Further analysis of the contours of group-2 reveals that the relative contact pressure at the edge and corner to the center were 54% (25/46) and 30% (14/46) for F1, 54% (25/46) and 30% (14/46) for F4, and 42% (24/57) and 10% (6/57) for F5. The outcome of (F4) test indicated that the upper grogrid layer (which is close to the footing) had no effect on the footing behavior, while the lower one was far enough to concentrate the stress below the load. As shown in Fig. 18, using one or two layers of geogrid almost did not affect the stress distribution below the geogrid layers. Finally, for group-3, the relative contact pressure at the edge and corner to the center 54% (25/46) and 30% (14/46) for F1, 45% (24/53) and 15% (8/53) for F6, and 35% (22/62) and 3% (2/62) for F7. These results assured that increasing the stiffness if the soil below the footing concentrates the stress below the load as reported by^13,14^.

### Modulus of subgrade reaction

**Fig. 19:**
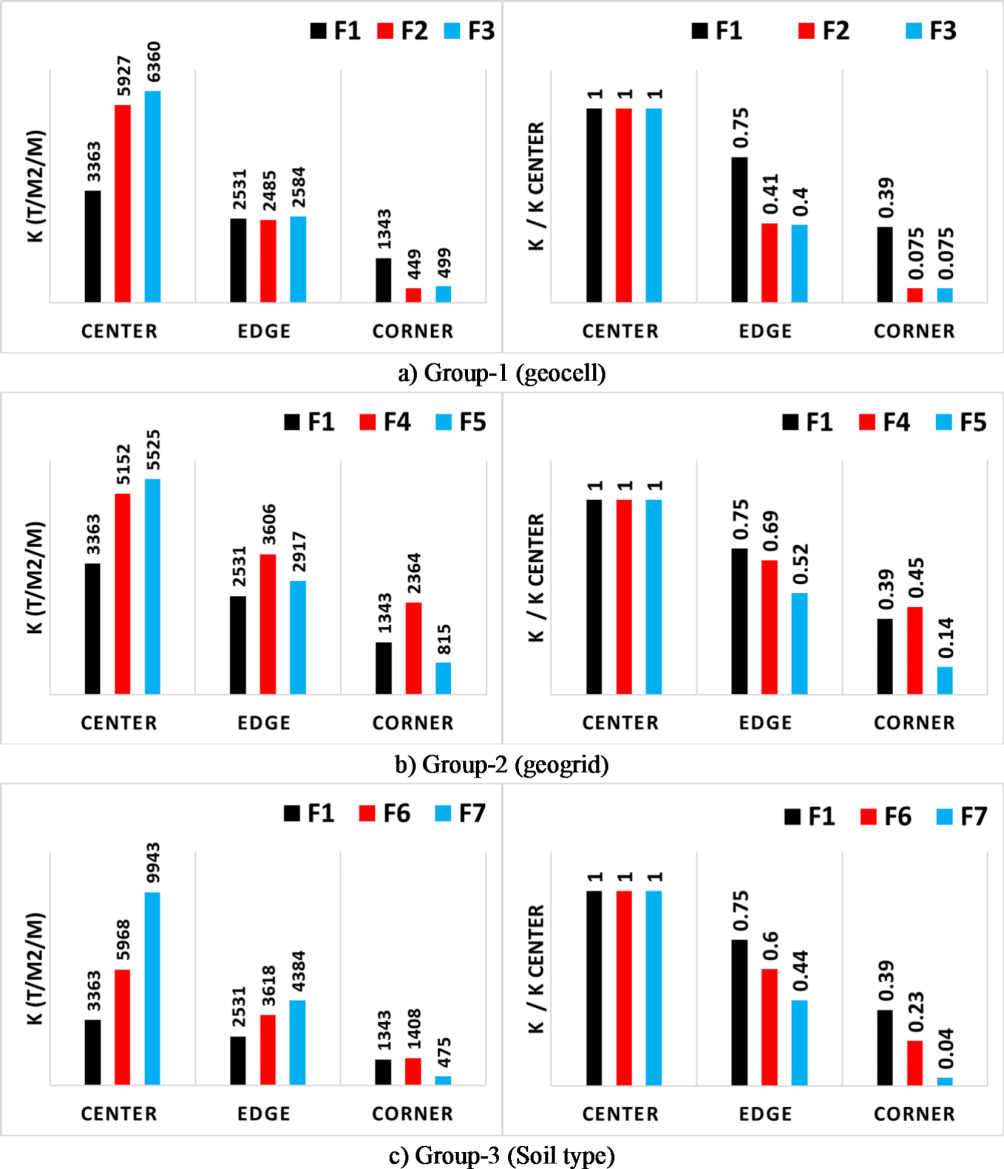
Absolute and relative (K) values at center, mid-edge and corner of footing.

The modulus of subgrade reaction (K) is the ratio between the contact pressure and the corresponding settlement (K = contact pressure/settlement). Calculating these values at the center, mid-edge and corner of the footing using the listed results in Table 2 leads to shown values in Fig. 19. It could be noted that using geocell layers increased the (K) value at the center by 75% and 90% for one and two layers in order, and reduced the (K) values at mid-edge and corner to 40% and 8% of the value at the center respectively. On the other hand, using geogrid enhanced the (K) value at the center by 55% and 65% for one and two layers respectively. While the reduction in the (K) values at mid-edge and corner were (70%, 45%) and (50%, 15%) of the value at the center for one and two layers in order. Finally, increasing the soil stiffness below the footing improved the (K) values at center by 200% and 80% for pure and mixed crushed stone in order. In addition, a corresponding reduction in the (K) values at mid-edge and corner to (45%, 5%) and (60%, 25%) of the value at the center. The results indicated that increasing the soil stiffness (by replacement, geogrid or geocell) increases the subgrade reaction value at the center and reduced it at edges and corners. This is because increasing the soil stiffness concentrated the stresses at the center (below the load) as shown in Fig. 17, while using geogrid and geocell uniformed that settlement below the footing^13,14^. Accordingly, (stress/settlement) increased at the center and reduced at the edges and corners.

## Conclusions

This study aims to investigate the impact of using geocell layers, geogrid layers and base soil stiffness on the punching capacity, settlement, contact pressure and subgrade reaction of an isolated footing. Seven identical footings were experimentally tested up to failure with continues recording of load, settlement, strain in rebars and contact pressure. The recorded measurements were analyzed, compared, discussed and graphically presented. The outcomes of this study could be summarized as follows:

- Based on the recorded strains in rebars, observed brittle failure and lower surface crack pattern, all the seven footings failed in pure punching.
- Generally, increasing the stiffness of the base granular soil below the footing either by confining (geocell), or by reinforcing (geogrid) or by replacing with stiffer soil concentrates the contact stress below the load. Accordingly, increases the punching capacity, subgrade reaction and reduces the settlement.
- All the three considered methods to increase the base granular soil increased the subgrade reaction at the center and reduced it at mid-edge and corner. This is due to concentrating the contact stress below the load (at the center) and reducing the settlement. However, the stress uniformity below the reinforcing layer is only achieved by using geogrid.
- The improvement due to using geocell and geogrid layers depends on how they are close to the footing. For geocell, the closest layer is the most efficient one. On the opposite hand, for geogrid, the closest layer is the least efficient one.
- The achieved results of using geocell layers, geogrid layers and granular soil replacement indicated increasing in punching capacity by (20%, 11% and 17%), and in subgrade reaction values at the center by(90%, 65% and 200%), and decreasing in settlement at the center by (70%, 75% and 45%) respectively.
- The observed improvement ratios in this study are limited by the considered geometric parameters and material properties.
- For farther studies, it is recommended to conduct a full numerical (FEM) parametric study verified by these results to develop more general prediction model for the punching capacity of isolated footing resting of geocell or geogrid layers.

## Data Availability

The datasets used and/or analysed during the current study available from the corresponding author on reasonable request.
